# Sex‐ and strain‐specific effects of mitochondrial uncoupling on age‐related metabolic diseases in high‐fat diet‐fed mice

**DOI:** 10.1111/acel.13539

**Published:** 2022-01-28

**Authors:** Leigh Goedeke, Kelsey N. Murt, Andrea Di Francesco, João Paulo Camporez, Ali R. Nasiri, Yongliang Wang, Xian‐Man Zhang, Gary W. Cline, Rafael de Cabo, Gerald I. Shulman

**Affiliations:** ^1^ Department of Internal Medicine Yale School of Medicine New Haven Connecticut USA; ^2^ Translational Gerontology Branch Intramural Research Program National Institute on Aging, NIH Baltimore Maryland USA; ^3^ Department of Physiology Ribeirao Preto School of Medicine University of Sao Paulo São Paulo Brazil; ^4^ Department of Cellular and Molecular Physiology Yale School of Medicine New Haven Connecticut USA

**Keywords:** 2,4‐dinitrophenol, anti‐aging, hepatic steatosis, insulin sensitivity, longevity, mitochondrial uncoupling

## Abstract

Mild uncoupling of oxidative phosphorylation is an intrinsic property of all mitochondria and may have evolved to protect cells against the production of damaging reactive oxygen species. Therefore, compounds that enhance mitochondrial uncoupling are potentially attractive anti‐aging therapies; however, chronic ingestion is associated with a number of unwanted side effects. We have previously developed a controlled‐release mitochondrial protonophore (CRMP) that is functionally liver‐directed and promotes oxidation of hepatic triglycerides by causing a subtle sustained increase in hepatic mitochondrial inefficiency. Here, we sought to leverage the higher therapeutic index of CRMP to test whether mild mitochondrial uncoupling in a liver‐directed fashion could reduce oxidative damage and improve age‐related metabolic disease and lifespan in diet‐induced obese mice. Oral administration of CRMP (20 mg/[kg‐day] × 4 weeks) reduced hepatic lipid content, protein kinase C epsilon activation, and hepatic insulin resistance in aged (74‐week‐old) high‐fat diet (HFD)‐fed C57BL/6J male mice, independently of changes in body weight, whole‐body energy expenditure, food intake, or markers of hepatic mitochondrial biogenesis. CRMP treatment was also associated with a significant reduction in hepatic lipid peroxidation, protein carbonylation, and inflammation. Importantly, long‐term (49 weeks) hepatic mitochondrial uncoupling initiated late in life (94–104 weeks), in conjugation with HFD feeding, protected mice against neoplastic disorders, including hepatocellular carcinoma (HCC), in a strain and sex‐specific manner. Taken together, these studies illustrate the complex variation of aging and provide important proof‐of‐concept data to support further studies investigating the use of liver‐directed mitochondrial uncouplers to promote healthy aging in humans.

## Graphical abstract

Here, we demonstrate that mild mitochondrial uncoupling via CRMP treatment reduces hepatic steatosis and improves hepatic insulin sensitivity in aged HFD‐fed mice. Long‐term CRMP treatment protects aged HFD‐fed mice against hepatocellular carcinoma in a strain and sex‐specific manner. Together, these studies illustrate the complex variation of aging and provide important proof of concept data to support further studies investigating the use of mitochondrial uncoupling agents to promote healthy aging in humans.

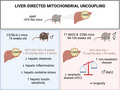

## 1 INTRODUCTION

Aging manifests as a gradual decline of organismal homeostasis that increases the risk for numerous disorders (e.g., cardiovascular disease, neurodegenerative disease, insulin resistance, diabetes, and cancer) and the capacity to survive (Baker & Peleg, 2017; Lopez‐Otin et al., 2013). Fueled by the increasing demographic of an aging population, research over the past years has focused on isolating associated factors that may broaden our understanding of the aging process and uncovering interventions that could significantly improve and increase human lifespan (Ros & Carrascosa, 2020; Samaras et al., 2014). Among these anti‐aging treatments, caloric restriction without malnutrition (CR) has consistently been shown to have beneficial effects on longevity and is the gold standard for interventions to promote healthy aging (Holloszy & Fontana, 2007). Unfortunately, there is a high degree of variability in the response to CR and most individuals have difficulty sustaining the strict dietary regime that is necessary to improve health and survival (Anderson et al., 2017). Accordingly, interventions that effectively mimic the benefits of CR (without limiting food intake) are being explored, including AMPK activators (metformin), inhibitors of GH/IGF‐1 axis (pegvisomant), inhibitors of mTOR (rapamycin), and activators of the sirtuin pathway (resveratrol; Son et al., 2019). Hormonal replacement in the elderly has also widely been used to improve various symptoms associated with aging; however, the possibility of side effects is a concern and new anti‐aging treatments that target fundamental processes of aging itself are desperately needed (Bornstein et al., 2020; Samaras et al., 2014).

Mitochondrial uncouplers are lipophilic, weak acids that transport protons across the inner mitochondrial membrane independently of ATP synthase, thereby uncoupling nutrient oxidation from ATP production and dissipating the proton gradient as heat (Childress et al., 2018). This results in greater proton influx into the matrix and a reduction in the mitochondrial membrane potential (↓+), which leads to diminished $O_{2}^{‐}$ production. The “uncoupling to survive” hypothesis (Brand, 2000) suggests that mitochondrial uncoupling will favor longevity by diminishing oxidative damage and improving mitochondrial function, an antagonistic hallmark of mammalian aging (Lopez‐Otin et al., 2013, 2016). Indeed, multiple studies in yeast, *Caenorhabditis elegans*, flies, rodents, and canines have shown that mild mitochondrial uncoupling reduces reactive oxygen species (ROS) production, delays the progression of age‐related diseases (i.e., hepatic steatosis and diabetes) and extends lifespan (Barros et al., 2004; Caldeira da Silva et al., 2008; Fridell et al., 2005, 2009; Lemire et al., 2009; Neretti et al., 2009; Nicholatos et al., 2019; Perry et al., 2013, 2015; Samuel et al., 2004, 2007; Ulgherait et al., 2020). Notably, treatment of mice with low‐dose 2,4‐dinitrophenol (DNP) enhances tissue respiratory rates, resulting in lower fasting plasma glucose, insulin, and lipid concentrations, reductions in oxidative stress, and enhanced longevity (Caldeira da Silva et al., 2008). While these studies suggest that mitochondrial uncoupling may be an efficacious anti‐aging therapy, the narrow therapeutic window of DNP precludes its use in the clinic.

In this regard, investigators have long been interested in identifying mitochondrial uncouplers with a wider safety margin. We previously developed a slow‐release formulation of DNP (controlled‐release mitochondrial protonophore, CRMP) that selectively increases hepatic mitochondrial oxidation by virtue of its first‐pass hepatic metabolism following oral ingestion (Perry et al., 2015). Due to its extended‐release coating, CRMP avoids peak (*C*_max_) plasma DNP concentrations, increasing the toxic to effective dose of DNP by more than 100‐fold and minimizing systemic exposure and hyperthermia (Perry et al., 2015). Unassociated with any detectable changes in body temperature, whole‐body energy expenditure, or body weight, oral delivery of CRMP safely reverses non‐alcoholic fatty liver disease (NAFLD), insulin resistance, diabetes, hepatic inflammation/fibrosis, and tumor growth in numerous dysmetabolic rodent models (Abulizi et al., 2017, 2019, 2020; Perry et al., 2015; Wang et al., 2018). Moreover, we recently found that CRMP is sufficient to reduce dyslipidemia, hepatic steatosis, and hepatic insulin resistance in spontaneously obese non‐human primates due to subtle sustained increases in hepatic mitochondrial oxidation (Goedeke et al., 2019). Collectively, our published studies provide important proof‐of‐concept data to support the development of liver‐directed mitochondrial uncouplers for the treatment of metabolic syndrome in humans; however, their effect on healthspan and lifespan remains to be determined. Here, we evaluated the sex‐ and strain‐specific effects of CRMP on high‐fat diet (HFD)‐associated metabolic disorders in aging mice. We hypothesized that long‐term liver‐directed mitochondrial uncoupling would reduce ROS production and increase longevity in mice, as well as improve insulin sensitivity, hepatic steatosis, and hepatic inflammation.

## 2 RESULTS

### 2.1 CRMP treatment is well tolerated and does not induce systemic toxicities in HFD‐fed aging male C57BL/6J mice

**FIGURE 1:**
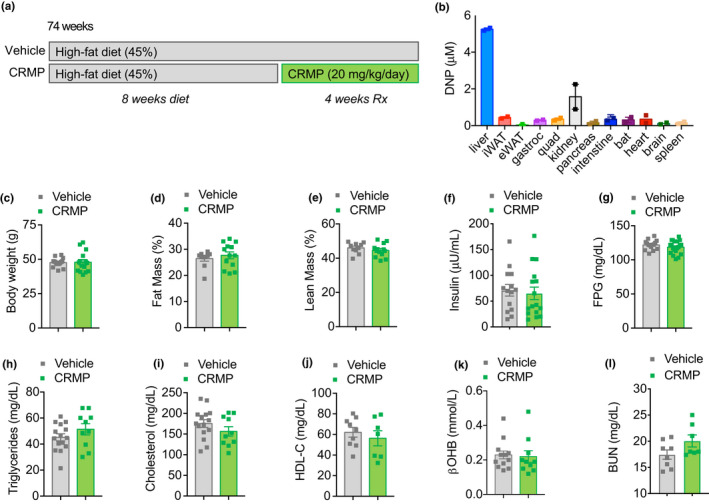
Effect of CRMP on metabolic syndrome in aged HFD‐fed male C57BL/6J mice. (a) C57BL/6J 74‐week‐old male mice were fed a high‐fat diet (HFD, 45% fat) for 8 weeks and then treated with CRMP (20 mg/[kg‐day]) or vehicle control for an additional 4 weeks. (b) Tissue concentrations of DNP in 6 h fasted CRMP‐treated mice. Body weight (c), % fat mass (d), % lean mass (e), plasma insulin (f), FPG (g), plasma triglycerides (h), plasma total cholesterol (i), plasma HDL‐C (j), β‐OHB (k), and BUN (l) in 6 h fasted aged male mice treated as in (a). In all panels, data are presented as mean ± SEM. *n* = 2 (b), 10–15 (c, h‐i), 10–13 per group (d‐e, k), 14–16 per group (f), 15–20 (g), 7–9 per treatment group (j), and 7–8 (l) per treatment group. β‐OHB, β‐hydroxybutyrate; CRMP, controlled‐release mitochondrial protonophore; DNP, 2, 4‐dinitrophenol; FPG, fasting plasma glucose; HFD, high‐fat diet

Excess caloric intake reduces longevity and increases the incidence of age‐related pathologies, including hepatic steatosis and insulin resistance (He et al., 2018; Nunes‐Souza et al., 2016; Weindruch & Sohal, 1997). To initially determine the effects of liver‐directed mitochondrial uncoupling in aged animals, 74‐week‐old C57BL/6J male mice were fed a HFD (45% fat) for 8 weeks to induce hepatic steatosis and insulin resistance and then treated with 20 mg/(kg‐day) CRMP or vehicle control for an additional 4 weeks (Figure 1a). A dose of 20 mg/(kg‐day) was chosen based on previous pharmacokinetic studies which demonstrated that 20 mg/kg‐day treatment resulted in hepatic DNP concentrations of ~5 μM (Figure 1b), concentrations that are slightly above that needed to promote a sustained increase in hepatic mitochondrial fat oxidation in rodents and non‐human primates (Goedeke et al., 2019; Perry et al., 2015). Consistent with previous studies (Abulizi et al., 2017, 2019, 2020; Perry et al., 2015), CRMP treatment did not significantly alter body weight, fat mass, or lean mass (Figure 1c–e). Whole‐body energy expenditure, food/water intake, activity, and RER were also unchanged between treatment groups (Figure S1), demonstrating the feasibility of using a controlled‐release formulation of DNP (CRMP) to avoid peak (*C*_max_) plasma DNP concentrations and prevent systemic toxicities and consistent with CRMP being liver‐directed. No appreciable differences were detected in fasting plasma glucose (FPG), insulin, triglycerides, total cholesterol, HDL‐C, β‐hydroxybutyrate (βOHB), or BUN concentrations (Figure 1f–k).

### 2.2 Liver‐directed mitochondrial uncoupling reduces hepatic lipid content and improves hepatic insulin sensitivity in aging HFD‐fed mice

**FIGURE 2:**
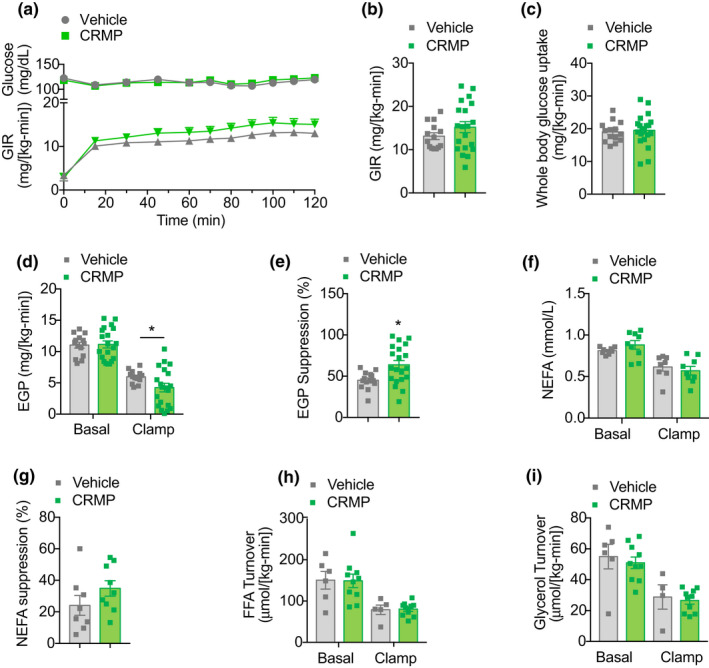
CRMP improves hepatic insulin sensitivity in aged HFD‐fed male C57BL/6J mice. (a–b) Plasma glucose and glucose infusion rate (GIR) during a hyperinsulinemic‐euglycemic clamp (3 mU/[kg‐min]) in aged HFD‐fed male C57BL/6J mice treated with CRMP (20 mg/[kg‐day]) for 4 weeks. (c) Whole‐body glucose uptake in aged male mice treated as in (a). (d) EGP during the basal and steady‐state period of the clamp. (e) Insulin‐mediated suppression of EGP during the clamp. (f) Plasma NEFA levels during the basal and steady‐state period of the clamp. (g) Insulin‐mediated suppression of plasma NEFAs during the clamp. Whole‐body FFA (h) and glycerol (i) turnover during the basal and steady‐state period of the clamp. In all panels, data are presented as mean ± SEM. *n* = 14–20 (a–e), 8–9 (f–g), and 6–10 (h–i) per treatment group. *p < .05 by two‐sided unpaired Student's *t* test compared to vehicle control. CRMP, controlled‐release mitochondrial protonophore; EGP, endogenous glucose production; FFA, free fatty acids; GIR, glucose infusion rate; HFD, high‐fat diet; NEFA, non‐esterified fatty acids

We have previously demonstrated that CRMP significantly improves insulin sensitivity in young dysmetabolic rodent models (Abulizi et al., 2017, 2019; Perry et al., 2015). To determine whether liver‐directed mitochondrial uncoupling affects whole‐body insulin action in aged mice, we performed hyperinsulinemic‐euglycemic clamps in HFD‐fed male mice treated with 20 mg/(kg‐day) CRMP for 4 weeks (Figure 1a). Under hyperinsulinemic conditions, the glucose infusion rate required to maintain euglycemia was similar between treatment groups (Figure 2a) and insulin‐mediated whole‐body glucose disposal was unchanged (Figure 2b). Importantly, hepatic DNP concentrations (~5 µM) were sufficient to protect aging HFD‐fed mice from hepatic insulin resistance, as insulin‐mediated suppression of endogenous glucose production (EGP) was significantly increased (Figure 2d–e). To assess whether CRMP reduced hepatic gluconeogenesis indirectly by altering white adipose tissue (WAT) lipolysis (Perry et al., 2015), we next measured plasma non‐esterified fatty acid (NEFA) concentrations and whole‐body fatty acid and glycerol turnover during the basal and hyperinsulinemic‐clamp period. As shown in Figure 2f–i, there were no significant differences in WAT lipolysis with CRMP treatment, suggesting that the CRMP‐induced improvements in hepatic insulin sensitivity in the aged mice were related to direct effects of CRMP on the liver.

**FIGURE 3:**
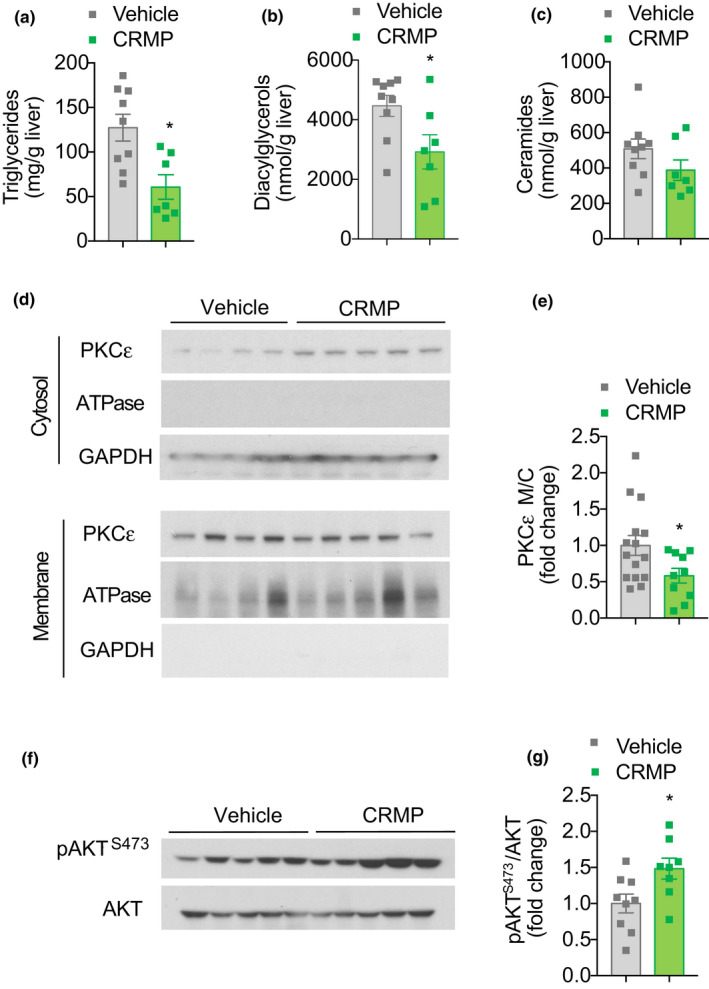
Liver‐directed mitochondrial uncoupling reduces hepatic ectopic lipid content in aged HFD‐fed male C57BL/6J mice. Hepatic triglyceride (a), diacylglycerol (b), and ceramide (c) content in aged HFD‐fed male C57BL/6J mice treated with CRMP (20 mg/[kg‐day]) for 4 weeks. (d) Representative Western blot of PKCε membrane to cytosol translocation in livers of aged mice treated as in (a). GAPDH and ATPase were used as loading controls. Quantification shown in panel (e). (f) Representative Western blot of pAKT^S473^/AKT in the livers of clamped aged mice treated as in (a). Quantification shown in panel (g). In all panels, data are presented as mean ± SEM. *n* = 7–9 (a–c), 10–15 (d–e), 10–13 (d–e), and 8–9 (f–g) per treatment group. *p < .05 by two‐sided unpaired Student's *t* test (a, e, g) or Mann–Whitney test (b) compared to vehicle control. CRMP, controlled‐release mitochondrial protonophore; HFD, high‐fat diet

Hepatic insulin resistance is closely associated with increased *sn*‐1,2 diacylglycerol content, translocation of protein kinase C epsilon (PKCε) to the plasma membrane and subsequent inhibition of insulin receptor kinase activity (Lyu et al., 2020; Petersen et al., 2016; Samuel et al., 2007; Ter Horst et al., 2017). To determine the mechanism by which CRMP treatment improved hepatic insulin sensitivity in HFD‐aged mice, we next measured ectopic lipid content and PKCε translocation in the livers of HFD‐fed aging mice treated with 20 mg/(kg‐day) CRMP for 4 weeks. Consistent with improved hepatic sensitivity, we found that CRMP treatment significantly reduced hepatic triglyceride and diacylglycerol (DAG) content by 40% (Figure 3a–b), while hepatic ceramide content was not significantly different between treatment groups (Figure 3c). Importantly, CRMP‐mediated decreases in hepatic DAG content were associated with a significant reduction in PKCε activity (as reflected by reduced PKCε membrane/cytosol translocation) and a significant ~2‐fold increase in insulin‐stimulated AKT phosphorylation (Figure 3d–f). Taken together, these data demonstrate that liver‐directed mitochondrial uncoupling is sufficient to reduce hepatic lipid content and improve hepatic insulin resistance in HFD‐fed aged mice.

### 2.3 CRMP treatment does not alter markers of hepatic mitochondrial biogenesis

Some of the beneficial effects of mild mitochondrial uncoupling have previously been attributed to activation of AMPK and alterations in mitochondrial biogenesis (Demine et al., 2019; Goedeke & Shulman, 2021). To determine whether CRMP‐mediated reductions in ectopic lipid content and improvements in hepatic insulin sensitivity were due to mitochondrial adaptations, we next evaluated the effects of CRMP treatment on several static markers of mitochondrial content in the livers of HFD‐fed aging mice treated with 20 mg/(kg‐day) CRMP for 4 weeks. As shown in Figure S2A, CRMP treatment did not significantly alter the mRNA expression of AMPK‐regulated genes involved in fatty acid oxidation (*Lcad*, *Mcad*, *Cpt1a*) or *de novo* lipogenesis (*Fasn*, *Acc1*, *Scd1*). Moreover, mRNA and protein expression of mitochondrial electron transport components were similar between treatment groups (Figure S2B–C). Hepatic mitochondrial DNA content, protein levels of the common mitochondrial markers (VDAC, PHB1, and COXIV), and the mRNA expression of genes involved in mitochondrial biogenesis were also unchanged (Figure S2D–F). Collectively, these studies suggest that CRMP‐mediated reductions in ectopic lipid content and improvements in hepatic insulin sensitivity were not due to alterations in hepatic mitochondrial content.

### 2.4 CRMP ameliorates hepatic inflammation and oxidative stress

**FIGURE 4:**
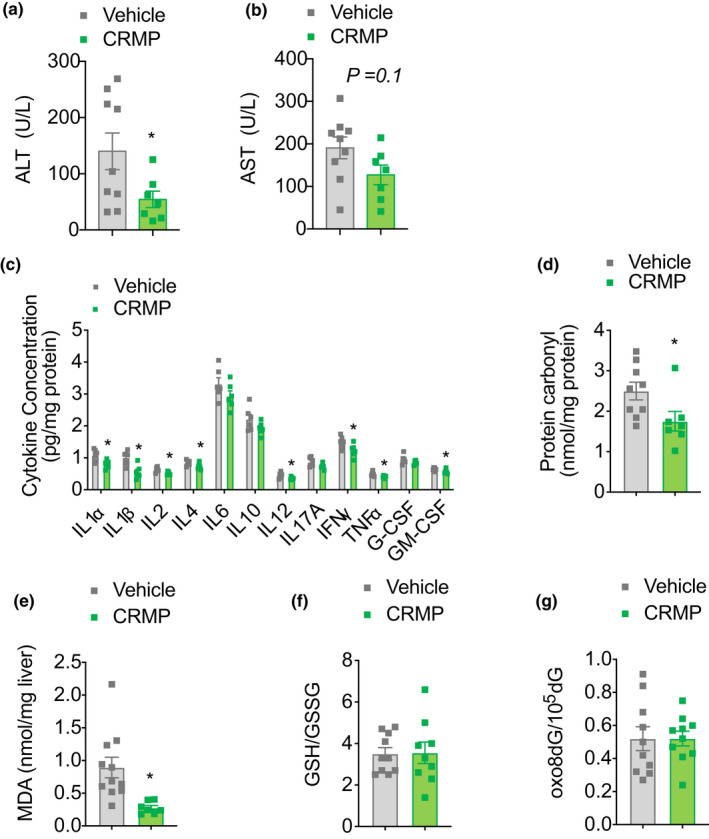
CRMP reduces hepatic inflammation and markers of oxidative stress in aged HFD‐fed male C57BL/6J mice. Plasma ALT (a), plasma AST (b), hepatic cytokine expression (c), hepatic protein carbonyl content (d), hepatic MDA (e), hepatic GSH/GSSG ratio (f), and hepatic oxo8dG/10^5^dG (g) in aged HFD‐fed male C57BL/6J mice treated with CRMP (20 mg/[kg‐day]) for 4 weeks. In all panels, data are presented as mean ± SEM. *n* = 7–9 (a–b, d), 6 (c), 7–11 (e), 9–10 (f), and 10 (g) per treatment group. *p < .05 by two‐sided unpaired Student's *t* test compared to vehicle control. CRMP, controlled‐release mitochondrial protonophore; HFD, high‐fat diet; MDA, malondialdehyde; GSH, glutathione; GSSG, glutathione disulfide; 8oxo8dG, 8‐Oxo‐7,8‐dihydro‐2′‐deoxyguanosine

Growing evidence links low‐grade, sustained hepatic inflammation and oxidative stress to the development of chronic liver disease, especially in the context of aging. As such, we next sought to investigate whether CRMP‐induced reductions in hepatic lipid content were associated with alterations in liver inflammation and oxidative stress in aging mice. As shown in Figure 4a–b, CRMP reduced plasma ALT by 60% (*p* < .05) and plasma AST levels by 40% (*p* = .1). Additionally, CRMP‐treated mice displayed a slight, but significant, reduction in the hepatic expression of several pro‐inflammatory cytokines, including IL1α, IL1β, IL2, IL4, IL12, IFNγ, TNFα, and GM‐CSF (Figure 4c). Moreover, CRMP significantly reduced several markers of hepatic oxidative stress, as reflected by the 30%–50% reduction in hepatic protein carbonyl content and lipid peroxidation (*p* < .05, Figure 4d–e), hallmarks of oxidative stress that have previously been linked with frail individuals (Fedorova et al., 2014; Ingles et al., 2014). Hepatic redox status (GSH/GSSG ratio) and DNA oxidation were not significantly altered with CRMP treatment (Figure 4f–g).

### 2.5 Sex‐ and strain‐specific effects of CRMP on metabolic changes in aging mice

**FIGURE 5:**
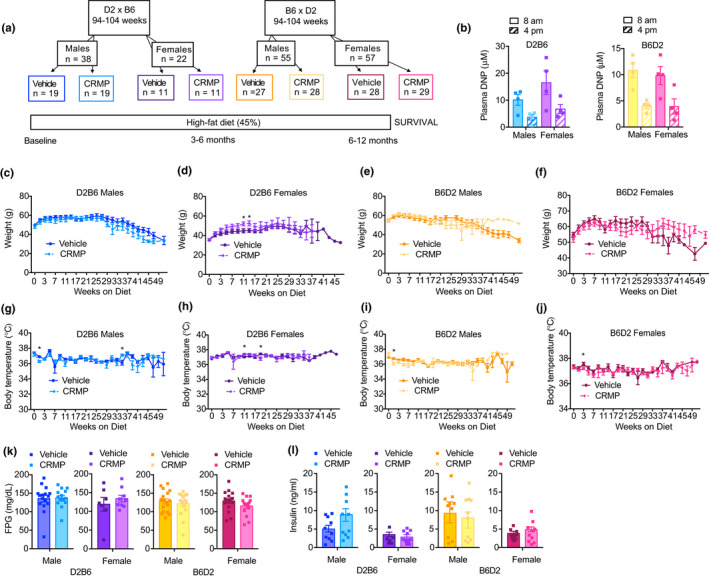
Sex‐ and strain‐specific effects of CRMP on age‐related metabolic disease in HFD‐fed mice. (a) Schematic diagram outlining long‐term aging study. 94–104 week‐old male and female F1 offspring of DBA/2J (D2) and C57BL/6J (B6) mice were fed a high‐fat diet (45% fat, HFD) or HFD containing CRMP (10 mg/[kg‐day]) for the remainder of their lives (~12 months). (b) Plasma DNP concentrations in 6 h fasted CRMP‐treated D2B6 and B6D2 mice. (c–j) Body weight (c–f) and body temperature (g–j) trajectories in D2B6 and B6D2 male and female mice. (K–L) 6 h FPG (K) and insulin (L) in D2B6 and B6D2 male and female mice treated for 3 months with CRMP (10 mg/[kg‐day]) or vehicle control. In all panels, data are presented as mean ± SEM. *n* = 4 (b), 11–29 (c‐j), 7–18 (k), and 7–11 (l) per treatment group. *p < .05 by two‐sided unpaired Student's *t* test compared to vehicle control (d, g–j). Abbreviations: C57BL/6J (B6); CRMP, controlled‐release mitochondrial protonophore; DBA/2J (D2); DNP, 2, 4‐dinitrophenol; FPG, fasting plasma glucose; HFD, high‐fat diet

A number of emerging experimental variables have been shown to influence healthspan and lifespan (Yuan et al., 2020). In particular, F1 hybrid strains produced by crossing C57BL/6J (B6) and DBA/2J (D2) mice respond to 40% CR in a maternal manner, demonstrating the importance of inherited mitochondrial pools in mitigating the beneficial effects of CR on lifespan extension and underscoring the importance of mitochondrial integrity during aging (Mitchell et al., 2016). Given the variables associated with the benefits of anti‐aging therapies, we next sought to test the effect of CRMP on healthspan and lifespan in two different F1 hybrid strains of mice. Specifically, male and female F1 progeny of B6 and D2 mice were fed a HFD (45% fat) or HFD containing 7 mg/g CRMP (~10 mg/[kg‐day]) starting at 94–104 weeks of age for the remainder of their lives (Figure 5a). In D2B6 and B6D2 male mice, ~3 months of CRMP treatment resulted in peak liver and plasma DNP concentrations of 5–10 µM, respectively (Figures 5b and S3A). Interestingly, B6D2 female mice achieved slightly higher hepatic DNP levels of 15 µM (Figure S3A), which could be explained by the increased food intake observed with CRMP treatment during this time (Figure S3B–E).

To investigate the sex‐ and strain‐specific metabolic changes induced by CRMP and HFD feeding during aging, we first measured body weight, body composition, and body temperature in D2B6 and B6D2 male and female mice. As expected, after 3 months of high‐fat feeding D2B6 and B6D2 mice gained weight (Figure 5c–f), due to an increase in fat mass and lean mass (Figure S4A–H). With advanced age (>25 weeks on diet), however, body weight began to decline in all treatment groups (Figure 5c–f). Interestingly, 3 months of CRMP treatment slightly, but significantly, increased lean mass in B6D2 males (Figure S4F), which has been associated with increased survival in rodent and human studies. Body temperature trajectories did not differ in all groups of mice throughout the course of the study (Figure 5c–j).

Aging is characterized by a general decline in cellular function, which ultimately affects whole‐body homeostasis (Lopez‐Otin et al., 2013). To determine whether CRMP treatment altered whole‐body metabolic function, we next assessed physical activity, whole‐body oxygen consumption, whole‐body carbon dioxide production, and respiratory exchange ratio (RER) in B6D2 and D2B6 mice treated with CRMP or vehicle control for 3 months. Indirect calorimetry revealed that CRMP treatment tended to increase whole‐body O_2_ consumption in female mice (Figure S5), consistent with B6D2 and D2B6 female mice consuming more CRMP and achieving higher plasma and liver DNP levels (Figure 5b and S3A). Spontaneous physical activity did not differ with age and high‐fat feeding or between treatment groups (Figure S5A–D). Interestingly, the RER tended to increase with CRMP treatment in B6D2 females and D2B6 males (Figure S5M–P), indicating a substrate preference toward glucose utilization; RER tended to decrease in D2B6 females (Figure S5N), indicative of increased fat oxidation. In contrast, there were no differences in fasting (6h) plasma glucose or insulin concentrations in mice treated with CRMP versus vehicle control for 3 months (Figure 5k–l).

### 2.6 CRMP reduces the incidence of neoplastic diseases in aging male B6D2 mice at the expense of non‐neoplastic disease

**FIGURE 6:**
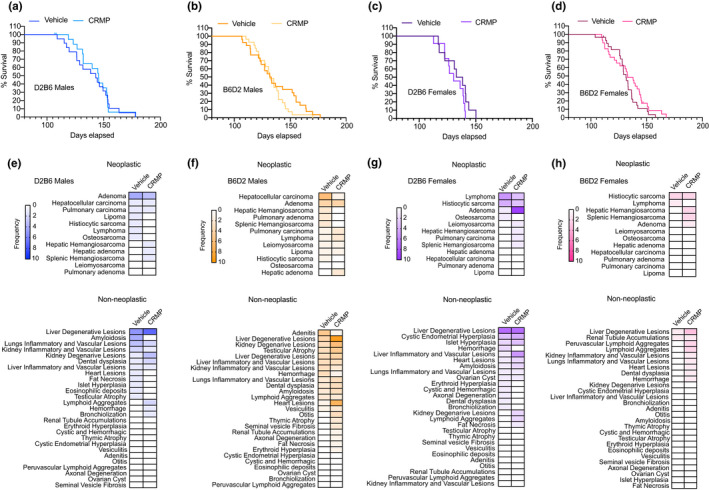
Effect of CRMP on neoplastic and non‐neoplastic disease incidence in HFD‐fed mice. Kaplan‐Meier survival curves for D2B6 males (a), B6D2 males (b), D2B6 females (c), and B6D2 females (d) fed a HFD (45%) or HFD containing CRMP (10 mg/[kg‐day]). (e–h) Heatmaps depicting frequency of neoplastic and non‐neoplastic disease incidence in mice treated as in (a–d). Mean age at necropsy: B6D2 males (35, 31 months); B6D2 females (32, 31 months); D2B6 males (30, 28 months); D2B6 females (27, 31 months). *n* = 30–31 per treatment group. C57BL/6J (B6); CRMP, controlled‐release mitochondrial protonophore; DBA/2J (D2); HFD, high‐fat diet

We next evaluated the impact of liver‐directed mitochondrial uncoupling on median and maximum lifespan in B6D2 and D2B6 mice. Survival curves were not significantly different among treatment groups by the log‐rank test regardless of sex and strain (Figures 6a–d and S6A–B, Table S1), despite CRMP‐treated D2B6 and B6D2 males displaying a slight increase in first quarter lifespan (Figure 6a–b). Mice that died spontaneously underwent necropsy and histopathological analysis by board‐certified veterinary pathologists blinded to the treatment group. In general, B6D2 mice tended to display more neoplastic pathologies upon examination than D2B6 mice (Figure 6e–h and Table S2), with male B6D2 mice exhibiting more prevalence for developing hepatocellular carcinoma (HCC), one of the most common lesions occurring in B6 mice with age (Mitchell et al., 2019). Interestingly, CRMP treatment significantly reduced the occurrence of HCC in male B6D2 mice at the expense of increased liver degenerative disease (*p* = .057; Table 1; Figure 6f), which could explain why CRMP‐treated B6D2 males did not live longer than vehicle controls. In contrast to male mice, female mice tended to display an overall increase in the number of neoplastic and non‐neoplastic pathologies with CRMP treatment (Figure 6g–h, Figure S5C–F); however, this did not reach significance (Table S2). Overall, these data highlight the sex‐ and strain‐specific differences in disease incidence and long‐term mitochondrial uncoupling, which likely result in distinct variation in healthy aging.

**TABLE 1:** Effect of CRMP treatment on chronic liver disease incidence

Disease	Males				Females			
	B6D2		D2B6		B6D2		D2B6	
	Vehicle	CRMP	Vehicle	CRMP	Vehicle	CRMP	Vehicle	CRMP
Mean age at necropsy (months)	35	31^§^	30	28	31	30	28	31
Neoplastic‐all	21	13*	10	8	11	21	3	7
Neoplastic‐liver	8	3*	1	2	0	2	0	2
Hepatic adenoma	0	2	0	0	0	0	0	0
Hepatocellular carcinoma	6	1^$#^	1	1	0	0	0	0
Hemangiosarcoma	2	0	0	2	0	2	0	2
Non‐Neoplastic‐all	37	62*	22	20	24	30	2	13
Non‐neoplastic‐liver								
Liver degenerative lesions	4	9*^#^	5	7	7	7	1	3
Lipidosis	1	3	2	3	1	5	1	2
Necrosis	3	6	3	4	6	2	0	1
Liver inflammatory and vascular lesions	3	3	1	1	1	4	0	0
Hepatitis	1	2	1	0	0	1	0	0
Telangiectasia	2	1	0	1	1	3	0	0
Vascular thrombosis	0	0	0	0	0	1	0	0

§ *p* < .05 by Student's *t* test (vehicle vs. CRMP).

* *p* < .05 by Fisher's exact test (neoplastic vs. non‐neoplastic disease).

$ *p* < .05 by Fisher's exact test (vehicle vs. CRMP).

# *p* = .057 by Fisher's exact test (Hepatocellular carcinoma vs. liver degenerative disease).

## 3 DISCUSSION

The quest to understand why we age has given rise to numerous lines of investigation that have gradually converged on the mitochondria as a major player in metabolic control (Lopez‐Otin et al., 2013; Mookerjee et al., 2010; Zimmermann et al., 2021). In particular, mitochondrial dysfunction, including reduced oxidative capacity and increased ROS production, has emerged as one of the main hallmarks of mammalian aging (Baker & Peleg, 2017; Bratic & Larsson, 2013; Chistiakov et al., 2014; Wang & Hekimi, 2015). Indeed, genetic mutations that dysregulate mitochondrial function clearly associate with accelerated aging phenotypes and increase susceptibility to disease (Bornstein et al., 2020; Vina et al., 2018; Warraich et al., 2020). Here, we sought to leverage the higher therapeutic index of CRMP (Perry et al., 2015) to test whether subtle increases in hepatic mitochondrial inefficiency could improve healthspan and lifespan in diet‐induced aged obese mice. We hypothesized that CRMP might increase longevity due to its ability to prevent the formation of ROS, as well as improve insulin sensitivity, hepatic steatosis, and hepatic inflammation.

To initially test our hypothesis, we examined the effect of CRMP treatment (20 mg/(kg‐day) × 4 weeks) on HFD‐associated metabolic disease in aged mice. Consistent with our previous studies in rodents and non‐human primates (Abulizi et al., 2017, 2019, 2020; Goedeke et al., 2019; Perry et al., 2013), daily oral delivery of CRMP was able to significantly reduce hepatic steatosis and improve hepatic insulin sensitivity due to alterations in hepatic DAG content and PKCε translocation. We also found evidence that subtle sustained increases in mitochondrial uncoupling could inhibit hepatic inflammation and lower hepatic protein carbonyl content and lipid peroxidation, markers of oxidative stress that have previously been linked with frail individuals (Ingles et al., 2014). Interestingly, CRMP did not significantly reduce fasting plasma triglycerides, fasting plasma glucose, or whole‐body insulin resistance in this dysmetabolic aging model, in contrast with several other younger HFD‐fed rodent models of obesity (Abulizi et al., 2017; Perry et al., 2013, 2015). As the mobilization of hepatic lipids is impaired in old mice (Araki et al., 2004), it is possible that age‐associated alterations in VLDL production may have prevented CRMP‐mediated reductions in plasma triglyceride concentrations and muscle ectopic lipid content, thus obstructing differences in insulin‐stimulated muscle glucose uptake. In agreement with this, we recently found that mild mitochondrial uncoupling was not sufficient to lower fasting plasma lipids and improve whole‐body insulin sensitivity in dysmetabolic mouse models with impaired triglyceride metabolism (L‐*Mttp* ^−/−^ and HSD11B1 Tg mice; Abulizi et al., 2019, 2020).

Given the beneficial effects of late‐stage CRMP intervention on HFD‐induced insulin resistance in aging C57BL/6J mice, we next sought to determine whether CRMP could improve and prolong the lifespan of male and female B6D2 and D2B6 mice subjected to 45% HFD at 94–104 weeks of age. Interestingly, liver‐directed mitochondrial uncoupling by CRMP had no beneficial effect on body weight, core body temperature, or insulin sensitivity; moreover, no significant differences were observed with treatment on median or maximal lifespan regardless of sex or strain. While plasma and liver DNP levels were in the therapeutic range after 3 months of treatment (which correlated with a trend toward increased first quartile lifespan extension in male D2B6 and B6D2 mice), it is possible that similar plasma/hepatic levels of DNP were not achieved by the end of the study due to aging‐accelerated declines in mitochondrial and OXPHOS dysfunction (Shpilka & Haynes, 2018). Indeed, biphasic alterations of mitochondrial activity have been proposed to occur with age, and the timing of mitochondrial manipulation with CRMP (94–104 weeks of age) may have been applied too late to counteract the burst in oxidative damage that occurs during middle age and persists through later stages of life (Baker & Peleg, 2017). In support of age‐dependent pleiotropy (Williams & Day, 2003), seemingly inconsistent results have emerged in relation to the causal role of mitochondrial (dys)function in aging (Dai et al., 2017; Wang & Hekimi, 2015). Indeed, Forster MJ et al demonstrated that CR, a robust metabolic and lifespan intervention associated with enhanced mitochondrial function and reductions in oxidative stress, had a deleterious effect on mortality when implemented in mice of advanced age (Forster et al., 2003). The pleiotropy associated with HFD and/or background strain may also have contributed to the lack of lifespan extension observed with CRMP treatment, as low‐dose DNP implemented at 18 weeks of age was highly effective at reducing oxidative stress, improving lipid metabolism, and extending lifespan in chow‐fed female Swiss Webster outbred mice (Caldeira da Silva et al., 2008). Intriguingly, the lard‐based HFD used in our study seemed to attenuate the aging phenotype seen in chow‐fed mice (Gutierrez‐Casado et al., 2019; Mitchell et al., 2016) and several studies have shown that lard‐based HFDs can prolong lifespan and mitigate age‐associated disorders, such as cardiovascular disease in mice (Oike et al., 2020; Poncelas et al., 2015).

Genetic background can also alter mitochondrial function and contribute to the complexity associated with cardiometabolic phenotypes in mice (Norheim et al., 2019). While we had initially designed our study to assess some of the sex‐gene interactions that may influence CRMP’s ability to increase longevity (female B6 mice have reduced response to 40% CR and altered expression of *Mdh2*, which affects the malate‐aspartate shuttle and increases cellular antioxidant function (Mitchell et al., 2016)), both F1 B6D2 and D2B6 cohorts carry loss‐of‐function mutations in the nicotinamide nucleotide transhydrogenase (*Nnt)* gene. This mutation could contribute to the differential effects on uncoupling‐mediated increases in longevity between our study and previous aging studies conducted with DNP (Caldeira da Silva et al., 2008), as the susceptibility to diet‐induced obesity is altered in the B6/J substrain with mutations in *Nnt* (Nicholson et al., 2010). Interestingly, we have tested the effects of CRMP in several rodent and non‐human primate diet‐induced obesity models which do not harbor mutations in the *Nnt* gene and find similar beneficial effects compared with those studies conducted on a B6/J background (Goedeke & Shulman, 2021). Alternatively, the beneficial effects of mitochondrial uncoupling by CRMP (i.e., reductions in hepatic lipid content/oxidative stress and improvements in hepatic insulin sensitivity) may simply not be sufficient to prolong lifespan in this model. Given that CRMP is liver‐directed, it is possible that mild systemic uncoupling is needed to regulate lifespan extension in mice. As such, future studies utilizing tissue‐specific uncouplers in chow and HFD‐fed mice on different backgrounds are warranted to understand the timing and underlying mechanisms surrounding the pleiotropic effects of mitochondrial dysfunction and how this translates to the potential therapeutic benefits of lifespan extension with mitochondrial uncoupling.

The liver undergoes numerous structural changes with increasing age, which ultimately lead to impaired liver function and increased risk for developing chronic liver disease (Calle & Kaaks, 2004; Calle et al., 2003). Chief among these is HCC, one of the most common neoplastic lesions occurring in HFD‐fed aging B6D2 and D2B6 mice (26% of all conditions, Table S2). Here, we find that long‐term CRMP treatment markedly reduced the occurrence of HCC in male B6D2 mice, presumably due to a reduction in hepatic oxidative stress (Sakurai et al., 2008) and the obesity‐enhanced production of IL‐6 and TNFα, which causes hepatic inflammation and activation of oncogenic STAT3 (Park et al., 2010), and which we have shown to be reduced in aging C57BL/6 male mice treated with CRMP for 4 weeks (Figure 4c–e). Interestingly, CRMP did not diminish the overall frequency of HCC in D2B6 F1 males, suggesting that genetic factors inherited from the female (i.e., mitochondrial differences) may modulate their resistance to the beneficial effects of CRMP on HCC incidence (Mitchell et al., 2016). Alternatively, the lower frequency of HCC in this mouse hybrid strain (2/18 male mice, 11%; Table S2) did not provide enough power to detect meaningful differences in HCC prevention.

Despite a greater reduction of HCC and other neoplastic conditions with CRMP treatment, B6D2 males did not live longer than vehicle‐treated controls. Similar findings have been found in other longevity studies (McCarter et al., 1997; Van Remmen et al., 2003), supporting a dissociation between cancer incidence and longevity in rodents. In particular, singly housed C57BL/6J mice were recently shown to display reduced cancer incidence compared to multiply housed mice, independently of lifespan extension (Ikeno et al., 2005). This was partially attributed to the increased incidence of non‐neoplastic disorders, analogous to our findings, which suggest male B6D2 mice were more vulnerable to other life‐threatening diseases due to a reduction in cancer. Alternatively, the significant differences in neoplasm incidence with CRMP treatment may be driven by age (mean age at necropsy for CRMP‐treated B6D2 males was 31 months, while vehicle‐treated mice were 35 months at necropsy; Table 1), as many mouse strains are rapidly dying of neoplastic disease after 30 months of age. Despite this, neoplastic disease is still highly prevalent starting around 24 weeks old, with neoplastic disease affecting 90% of mice at 30 months (Blackwell et al., 1995; Brayton et al., 2012). As such, the age differences observed at necropsy between CRMP and vehicle‐treated B6D2 mice were more likely due to the subsequent increase in non‐neoplastic disorders that occurred with CRMP treatment.

Collectively, these studies demonstrate that short‐term (4 weeks) CRMP treatment reduces hepatic steatosis, hepatic insulin resistance, and hepatic inflammation in aged HFD‐fed mice, independently of changes in body weight, whole‐body energy expenditure, food intake or markers of hepatic mitochondrial biogenesis. While this suggests that liver‐directed mitochondrial uncoupling agents may be beneficial in reducing metabolic syndrome in aged individuals, there was neither a lifespan nor net pathology benefit associated with CRMP and HFD treatment initiated late in life (2‐year‐old mice, ~70 years human equivalent). Our studies therefore highlight the complex variation of aging and support the age‐dependent pleiotropy of mitochondrial oxidative damage (Baker & Peleg, 2017). They also suggest that targeting the pathological levels of mitochondrial ROS in old age may offer better translational potential against metabolic disorders than life‐long interventions (Vina et al., 2018; Warraich et al., 2020). Indeed, liver‐directed mitochondrial uncoupling was able to significantly reduce HCC in B6D2 mice, only at the expense of increased non‐neoplastic disease. As proton leak may also display antagonistic pleiotropy in different tissues, the beneficial effects of uncoupling seen in the liver may not translate to other organ systems (Bellanti et al., 2013; Chiao et al., 2020; Serviddio et al., 2007). In support of this, it was recently shown that old mouse hearts display increased basal respiration and elevated proton leak, while normalization of proton leak in cardiomyocytes was protective against age‐induced cardiac dysfunction (Chiao et al., 2020). Future tissue‐specific studies are therefore warranted to assess the outcomes of subtle sustained increases in mitochondrial uncoupling on healthy aging in rodents. The development of mitochondrial‐targeted and tissue‐specific uncoupling agents promises to broaden our understanding of the aging process and highlight the potential role of tissue‐specific mitochondrial uncoupling agents as novel anti‐aging therapies.

## 4 EXPERIMENTAL PROCEDURES

### 4.1 Animals

Male C57BL/6J mice (aged 72 weeks) were purchased from Jackson Laboratory and kept under constant temperature and humidity in a 12 h controlled light/dark cycle (7 AM–7 PM). All mice were allowed to acclimate to the Yale Animal Resource Center for at least 1 week prior to any experimental use. Mice were multiply housed (3–4/standard cage) and fed a standard chow diet prior to experimentation. Chlorinated water was provided in an automatic watering system or water bottles. For HFD studies, 74‐week‐old mice were placed on a 45% HFD (45% HFD, D12451 Research Diets, Inc.) for 8 weeks and treated with 20 mg/(kg‐day) of CRMP in ~100 mg of peanut butter (Skippy^®^ Brand) or vehicle control between 0800 and 1000 h for an additional 4 weeks. Mice were singly housed during the 4‐week treatment period to ensure adequate consumption of the CRMP peanut butter mixture. Surgeries were performed under isoflurane anesthesia to place polyethylene catheters in the carotid artery (sampling) and jugular vein (infusion); the catheters were externalized on the back (caudal to the scapulae) with a dual‐channel vascular access button (Instech Labs), after which the catheters were locked with heparinized saline (200 U/ml; Shi et al., 2018). Carprofen analgesia was provided during the post‐operative period and singly housed mice were allowed to recover for 1 week prior to experimentation; only mice that recovered pre‐surgery body weight were studied. Mice were sacrificed by intravenous pentobarbital and tissue samples were collected as previously described and stored at −80°C until subsequent analyses (Perry et al., 2015). All animal studies were approved by the Institutional Animal Care and Use Committee of Yale University.

For survival studies, male and female C57BL/6J and DBA2/J mice were obtained from Jackson Laboratory and housed at the National Institute on Aging. Mice were bred to generate F1 B6D2F1/J (B6D2) and D2B6F1/J (D2B6) male and female mice as previously described (Mitchell et al., 2016). Mice were fed standard chow (2018 Teklad Global 18% Rodent Diet, Harlan Teklad) ad libitum until 94–104 weeks of age, at which point mice were randomized into two treatment groups: vehicle (45% HFD, D12451 Research Diets, Inc.) or CRMP (45% HFD +7.5 mg CRMP/g diet, D17061502 Research Diets, Inc.) for the remainder of their lives (*n* = 19 [D2B6 males], 11 [B6D2 females], 27–28 [B6D2 males] or 28–29 [B6D2 females] per treatment group). Body weight, food intake, and body temperature were monitored biweekly as previously described (Mitchell et al., 2016). Body composition and fasting clinical chemistry were assessed as described below. All animal protocols were approved by the National Institute on Aging Animal Care and Use Committee.

### 4.2 Survival study and pathology

Throughout the course of the longevity, study animals were inspected twice daily for health issues and deaths were recorded for each animal. Moribund animals were euthanized based on an independent assessment by veterinarians according to the AAALAC guidelines, and only cases, where the condition of the animal was considered incompatible with continued survival, are represented as deaths in the curves. A random selection of mice that died as part of the study was subject to gross histopathological analysis (*n* = 31 vehicle and 30 CRMP). Organs were collected and fixed in 4% PFA for further analysis for pathology based on board‐certified histopathologists blinded to treatment groups (Mitchell et al., 2016). Two mice were censored to determine hepatic DNP levels after 3 months of treatment (1 B6D2 female and 1 B6D2 male).

### 4.3 Whole‐body metabolic assessment

Whole‐body metabolic assessment was performed at Yale University (male C57BL/6J mice) and the National Institute on Aging (male and female B6D2 and D2B6 mice) using the Comprehensive Lab Animal Monitoring System (CLAMS; Columbus Instruments). Cage activity, energy expenditure, oxygen consumption, carbon dioxide production, and food consumption were recorded for a 48 h period and normalized to lean body mass (Figure S1) or body weight (Figure S4). Drinking was assessed by a computer system counting consumed water droplets. Fat and lean mass were assessed by ^1^H‐magnetic resonance spectroscopy (Bruker BioSpin). Rectal temperature was recorded with a BAT‐12 Microprobe Thermometer with at RET3 rectal probe for mice (Physiotemp Instruments, Inc.) as previously described (Mitchell et al., 2016).

### 4.4 Hyperinsulinemic‐euglycemic clamp experiments

Hyperinsulinemic‐euglycemic clamps were performed as previously described with several modifications (Perry et al., 2015; Shi et al., 2018). On the day of the clamp experiment, mice were placed in a clean cage with bedding and fasted for 6 h starting at 7 AM. At *t* = −180 min, the catheters were flushed with heparinized saline (10 U/ml) and the mice were connected to the infusion lines. Whole‐body glucose, fatty acid, and glycerol turnover were assessed by [3‐^3^H] glucose (HPLC purified; Perkin‐Elmer Life Sciences), [1,1,2,3,3‐d_5_] glycerol (Sigma‐Aldrich) and [^13^C_16_] sodium palmitate (Cambridge Isotopes) infused at rates of 0.05 μCi/min, 1.5 μmol/(kg‐min), and 300 mg/(kg‐min), respectively, into the jugular catheter starting at *t* = −120 min. The clamp was initiated at *t* = 0 min with a continuous infusion (3.0 mU/(kg‐min) of human insulin (Novolin; Novo Nordisk) and washed red blood cells (obtained from a donor mouse) to compensate for blood loss due to repeated sampling (5 μl/min of 50% RBC in 10 U/ml heparinized saline). [1,1,2,3,3‐d_5_]glycerol (Sigma‐Aldrich) and [^13^C_16_] sodium palmitate (Cambridge Isotopes) continued to be infused at the above rates to assess whole‐body free fatty acid and glycerol turnover during the clamp period. Blood glucose was measured every 10–15 min (YSI Biochemistry Analyzer) and euglycemia was maintained by adjusting a variable infusion of 50% dextrose containing 0.06 μCi/μl of [3‐^3^H]glucose tracer. To assess tissue‐specific glucose uptake, a 10 μCi bolus of 2‐deoxy‐d‐[1‐^14^C]glucose (PerkinElmer) was injected at 85 min. Plasma samples were obtained from the venous catheter at 0, 15, 30, 45, 60, 70, 80, 90, 100, 110, and 120 min and processed for [3‐^3^H]glucose specific activity and palmitate and glycerol turnover as previously described (Perry et al., 2015). At the end of the clamps, mice were euthanized with a pentobarbital sodium injection (150 mg/kg). Tissues were taken, snap frozen in liquid nitrogen, and stored at −80°C for subsequent use.

### 4.5 Biochemical analysis

Blood samples were collected through arterial catheters after a 6 h fast unless otherwise noted. Blood was immediately placed in heparinized‐lithium tubes, separated by centrifugation, and stored at 4°C or −80°C for long‐term storage. Plasma glucose concentrations were measured using the YSI Biochemistry Analyzer or Ascensia Elite glucose meter (survival studies; Martin‐Montalvo et al., 2013); plasma insulin was measured by RIA or ELISA. Plasma NEFAs, total cholesterol, and HDL‐C concentrations were measured using enzymatic kits from Wako according to the manufacturer's instructions. Plasma triglycerides were measured using the Sekisui Triglyceride‐SL Kit (Sekisui Diagnostics) and plasma βOHB, AST, ALT, and BUN by COBAS (Roche Diagnostics).

### 4.6 Hepatic lipid and cytokine measurements

Liver triglycerides were extracted by the method of Bligh and Dyer in 6 h fasted mice and quantified using the Sekisui Triglyceride‐SL Kit as previously described (Petersen et al., 2016). Liver DAGs were extracted from the cytosolic‐ and membrane‐associated fractions and measured by LC‐MS/MS as described (Petersen et al., 2016). Total DAGs are reported as the sum of individual species. Liver ceramides were extracted from 6 h fasted mice and measured by LC‐MS/MS according to previously established methods (Petersen et al., 2016). Hepatic cytokines were measured by ELISA (Qiagen) as previously described (Abulizi et al., 2017).

### 4.7 Oxidative stress measurements

Protein carbonylation and lipid peroxidation were determined in the livers (~50 mg) of aged C57BL/6J mice after 4 weeks of CRMP treatment using the Protein Carbonyl Colorimetric Assay Kit (Cayman Chemical) and TBARS Assay Kit (Cayman Chemical), respectfully, according to the manufacturers’ instructions. Hepatic redox state (GSH/GSSG ratio) was determined by LC‐MS/MS as we have previously described (Madiraju et al., 2014).

Oxidative damage to DNA was measured as the level of 8‐oxo‐2‐deoxyguanosine (oxo8dG) using LC‐MS/MS as previously described (Jeng et al., 2015; Van Remmen et al., 2003). Hepatic nuclear DNA was isolated by NaI extraction using the DNA Extractor WB kit (Wako Chemicals). Briefly, liver (50–100 mg) was homogenized in ice‐cold lysis solution containing 0.15 mM deferoxamine (Sigma) with a Dounce homogenizer. Nuclei were collected by centrifuging the homogenate at 10,000* g* for 20s, and the nuclear pellets were resuspended in the enzyme reaction solution and proteinase K (10 μg/ml) provided with the kit. Following incubation at 50°C for 20 min, RNase cocktail (Ambion) was added to a final concentration of 20 μg/ml and samples were incubated for another 10 min at 50°C. After centrifugation at 10,000* g* for 5 min, the supernatant was collected and 0.3 ml of NaI solution was added. Samples were mixed by inversion and 0.5 ml isopropyl alcohol was subsequently added and homogenized by inversion until a whitish precipitate appeared. The precipitate was collected by centrifugation at 10,000* g* for 10 min at room temperature and washed with 1 ml washing solution A and B according to the manufacturer's instructions. After an additional centrifugation at 10,000 *g* for 5 min, residual liquid was removed and the resultant pellet was briefly dried at RT for 3 min. The DNA pellet was then solubilized in water containing 0.1 mM deferoxamine. DNA concentration was measured spectrophotometrically at 260 nm, and its purity was assessed by ensuring A260/A280 >1.7.

DNA hydrolysis was performed as described (Jeng et al., 2015) with some modifications. Briefly, nDNA samples (10–20 μg) were spiked with 2.82 pmol [^15^N_5_]‐8‐oxodGuo and 84.3 pmol of [^15^N_5_]‐dG (Cambridge Isotope Laboratories, Inc.). Then, 5 µl of 0.2 U/μl nuclease P1 (in 300 mM sodium acetate and 1 mM ZnSO_4_, pH 5.3) was added to the DNA solutions and the DNA was incubated for 1 h at 37°C. After this, 10 μl of 10× alkaline phosphatase buffer (500 mM Tris/HCl, pH 8, 1 mM EDTA) together with 4 µl of alkaline phosphatase was added and the incubated continued at 37°C for an additional 1 h. Subsequently, 10 μl of 0.1 M HCl was added to neutralize the solution. The 8‐oxo‐2‐deoxyguanosine (oxo8dG) and 2′‐deoxyguanosine (dG) concentrations were quantified by LC‐MS/MS as previously described (Jeng et al., 2015). The data are expressed as the ratio of nmol of oxo8dG to 10^5^ nmol of dG.

### 4.8 Plasma and tissue DNP measurements

For the longevity study, blood samples were drawn from non‐fasted male and female D2B6 and B6D2 mice at 8 AM and 4 PM. All other plasma and tissue DNP measurements were collected from C57BL/6J and D2B6 mice after a 6 h fast. DNP concentrations were measured in the plasma and tissues by LC/MS‐MS as previously described (Perry et al., 2015).

### 4.9 Gene expression and mtDNA analysis

Total mouse liver RNA was isolated using TRizol reagent (Invitrogen) and the RNEasy Mini Kit (Qiagen) according to the manufacturer's protocol. One microgram of total RNA was reverse transcribed to cDNA using the QuantiTect Reverse Transcription Kit (Qiagen), and qPCR was performed in duplicate using iTaq SYBR Green (Bio‐Rad) on an Applied Biosystems 7500 Fast qRT‐PCR System. Standard curves with pooled cDNA were used to calculate amplification efficiencies, and relative gene expression was normalized to 18S. mtDNA content was assessed as previously described (Price et al., 2012). Briefly, total DNA was extracted using the DNeasy blood and tissue kit (Qiagen) and mtDNA was amplified using primers specific for mitochondrial cytochrome c oxidase, subunit 2 (*Cox2*). mtDNA content was normalized to genomic DNA using primers specific for *Rps18*. Primer sequences are available upon request.

### 4.10 Western blotting

Liver lysates were prepared in RIPA buffer with freshly added protease inhibitors (cOmplete MINI; Roche) and phosphatase inhibitors (PhosSTOP; Roche) as previously described (Goedeke et al., 2018). After normalizing for equal protein concentration by the BCA assay (Pierce), lysates were resuspended in Laemmli sample buffer containing 2% β‐mercaptoethanol, boiled, and separated on 4%–8% or 10%–20% Tris‐Glycine Gels (Novex). Following a 2 h semi‐dry transfer onto PVDF membranes (Millipore), the membranes were blocked with 5% BSA (w/v) in wash buffer and probed with the following antibodies overnight at 4°C: pAKT^S473^ (Cell Signaling #4060; 1:1000), AKT (Cell Signaling #5239; 1:1000), HSP90 (BD Biosciences #610418; 1:1000), HSP60 (Cell Signaling #12165; 1:1000), VDAC (Cell Signaling #4661; 1:1000), PHB1 (Cell Signaling #2426; 1:1000), or COXIV (Abcam #ab16056; 1:1000). For analysis of ETC components, lysates were prepared in Laemmli sample buffer and separated on a 10%–20% Tris‐Glycine Gel (Novex). Following a 2 h semi‐dry transfer onto PVDF membranes, the membranes were blocked with 5% non‐fat milk (w/v) in wash buffer and probed with the following antibody cocktail overnight at 4°C: total OXPHOS (Abcam #ab110413; 1:1000). After washing in wash buffer, all membranes were incubated for 1 h at RT with HRP‐conjugated secondary antibodies (1:5000) diluted in blocking buffer. Bands were visualized by enhanced chemiluminescence (Pierce), and densitometry analysis was carried out using ImageJ software (NIH).

PKCε translocation was assessed in the livers of 6 h fasted mice using ultracentrifugation as we have previously described (Petersen et al., 2016) and lysates were subject to Western blotting as described above with the following antibodies: PKCε (BD #610086; 1:1000), GAPDH (Cell Signaling, #5174; 1:2000), and Na‐K ATPase (Abcam #ab7671; 1:500). The ratio of membrane PKC intensity (normalized to Na‐K ATPase) to cytosolic PKC intensity (normalized to GAPDH) was calculated.

### 4.11 Statistics

Data are expressed as mean ± SEM. Normality was checked using the Shapiro–Wilk normality test. Statistical differences were measured using an unpaired or paired two‐sided Student's *t* test, Wilcoxon matched‐pairs signed‐rank test, or one‐way ANOVA with Bonferroni corrections for multiple comparisons where appropriate. The effect of treatment on the frequencies of the presumptive causes of death was determined by chi‐square or Fisher's exact test where appropriate. A value of *p* ≤ .05 was considered statistically significant. Survival curves were compared by log‐rank tests. Data analysis was performed using GraphPad Prism software version 9 (Graphpad).

## CONFLICT OF INTEREST

GIS is an inventor on Yale patents for liver‐targeted mitochondrial uncoupling agents and controlled‐release mitochondrial uncoupling agents for the treatment of NAFLD, NASH, T2D, and related metabolic disorders and is a scientific co‐founder of TLC, Inc. GIS serves on the advisory boards for Merck, Novo Nordisk, AstraZeneca, Gilead Sciences, and Janseen Research and Development. GIS receives investigator‐initiated support from AstraZeneca, Gilead Sciences, and Merck. LG is a scientific advisor for TLC, Inc. The other authors declare that they have no competing interests.

## AUTHOR CONTRIBUTIONS

LG, JPC, RdC, and GIS designed the study. LG, KNM, ADF, ARN, YW, X‐MZ, and GWC performed the experiments. LG, KNM, ADF, GWC, RdC, and GIS analyzed and interpreted the data. LG and GIS wrote the manuscript with input from all authors.

## Supporting information

Supinfo S1

Table S2

## Data Availability

The data that support the findings of this study are available in the Supplementary Material of this article.
