# Supplementary material for: Sex‐ and strain‐specific effects of mitochondrial uncoupling on age‐related metabolic diseases in high‐fat diet‐fed mice
Source: Aging Cell. 2022 Jan 28;21(2):e13539. doi: 10.1111/acel.13539 (PMC8844126; doi:10.1111/acel.13539)
Supplement: Supplementary file 1 — Supinfo S1 [file ACEL-21-e13539-s002.pdf]

SUPPORTING INFORMATION

Sex- and Strain-Specific Effects of Mitochondrial Uncoupling on Age-Related Metabolic Diseases in High-Fat Diet-Fed Mice

Running title: Effect of CRMP on Metabolic Disease in Aging Mice

Leigh Goedeke[1], Kelsey N. Murt[2], Andrea Di Francesco[2], João Paulo Camporez[1,3], Ali R. Nasiri[1], Yongliang Wang[1], Xian-Man Zhang[1], Gary W. Cline[1], Rafael de Cabo[2], Gerald I. Shulman[1,4*]

Departments of [1]Internal Medicine and [4]Cellular & Molecular Physiology
Yale School of Medicine, New Haven CT 06520, USA
[2]Translational Gerontology Branch, Intramural Research Program,
National Institute on Aging, NIH, Baltimore, MD 21224, USA
[3]Department of Physiology, Ribeirao Preto School of Medicine,
University of Sao Paulo, Brazil

*To whom correspondence should be addressed: gerald.shulman@yale.edu

This file contains **Supplementary Figures 1–6** and **Supplementary Table 1**.

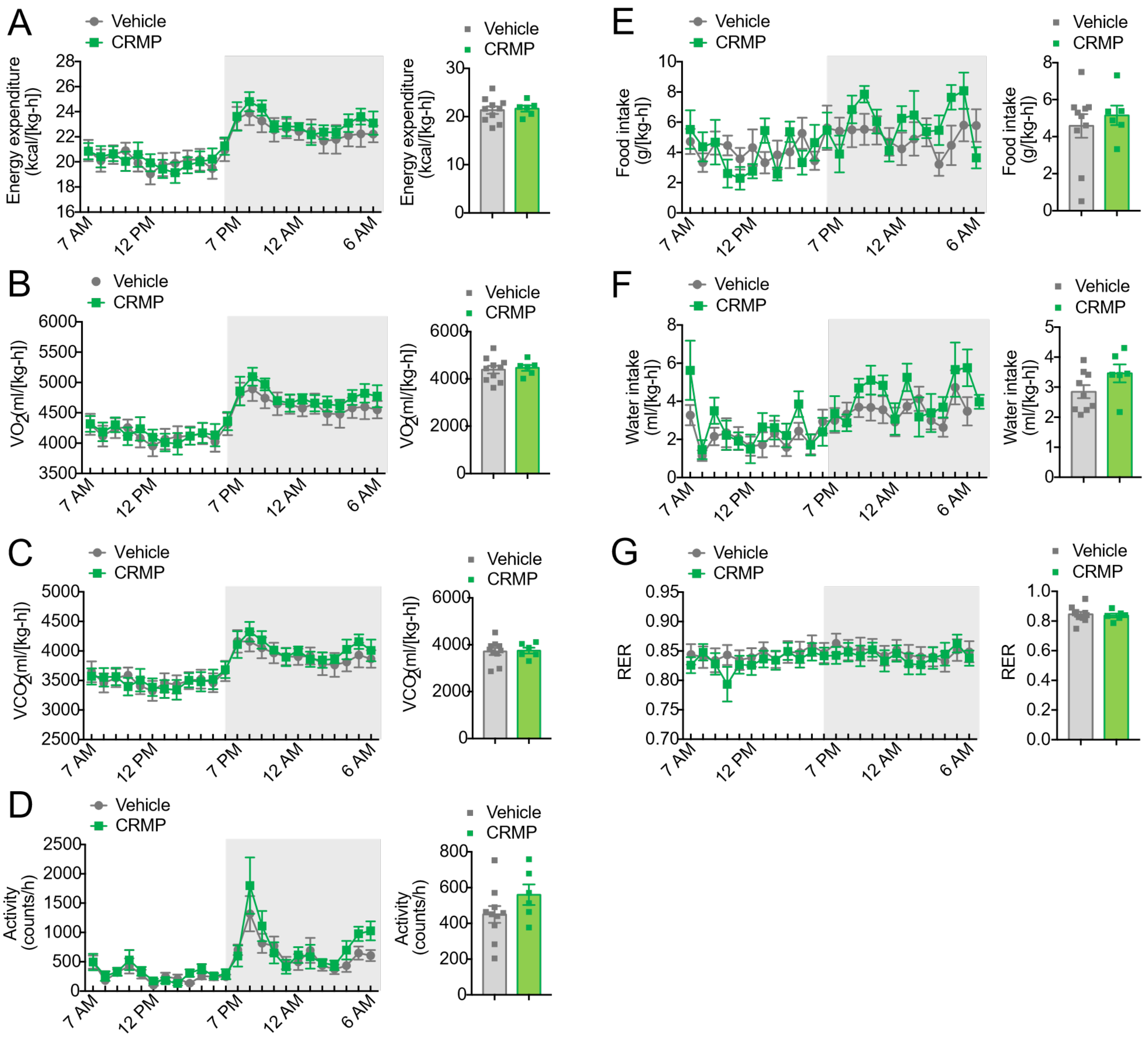

Figure S1: Metabolic parameters of aged HFD-fed male C57BL/6J mice treated with CRMP. (**A**–**D**) Whole-body energy expenditure (**A**), whole-body O_2 consumption (**B**), whole-body VO_2 production (**C**), locomotor activity (**D**), food intake (**E**), water intake (**F**), and RER (**G**) in 74-week-old male C57BL/6J mice fed a high-fat diet (HFD, 45% fat) for 8 weeks and then treated with CRMP (20 mg/[kg-day]) or vehicle control for an additional 4 weeks. In all panels data are presented as mean +/- SEM. *n* = 6–10 per treatment group. Abbreviations: CRMP, controlled-release mitochondrial protonophore; HFD, high-fat diet; RER, respiratory exchange ratio.

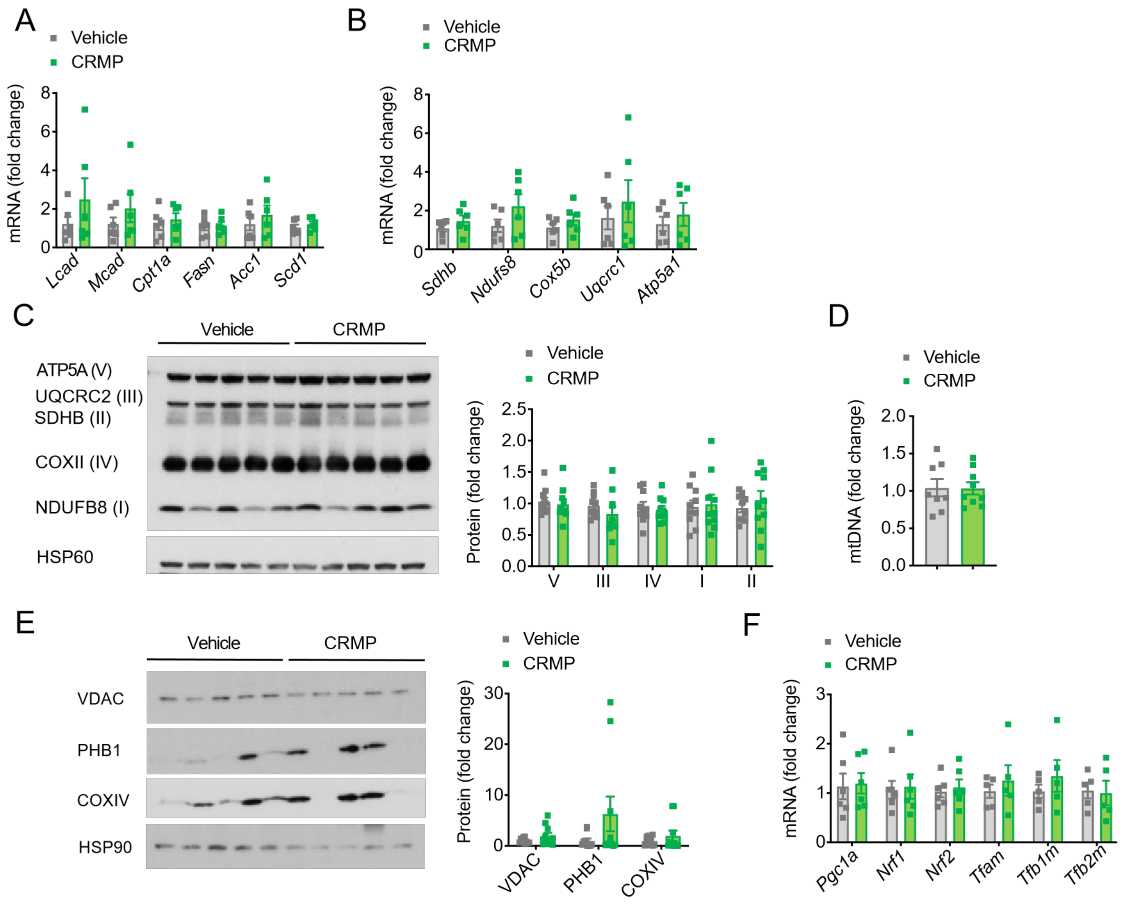

Figure S2: CRMP treatment does not alter hepatic fatty acid metabolism gene expression or markers of mitochondrial mass in aged HFD-fed male C57BL/6J mice. (**A**) qRT-PCR analysis of fatty acid metabolism genes in the livers of 74-week-old male C57BL/6J mice fed a high-fat diet (HFD, 45% fat) for 8 weeks and then treated with CRMP (20 mg/[kg-day]) or vehicle control for an additional 4 weeks. (**B–C**) qRT-PCR and representative Western blot analysis of mitochondrial ETC components in the livers of mice treated as in (**A**). HSP60 was used as a loading control. Quantification of blot shown to the right. (**D**) Mitochondrial DNA content in the livers of mice treated as in (**A**). **(E)** Representative Western blot analysis of mitochondrial markers in the livers of mice treated as in (**A**). HSP90 was used as a loading control. Quantification of blot shown to the right. (**F**) qRT-PCR analysis of *Pgc1a* targets in the livers of mice treated as in (**A**). In all panels data are presented as mean +/- SEM. *n* = 6 (**A–B**; **F**), 10 (**C, E**) or 8-9 (**D**) per treatment group. Abbreviations: CRMP, controlled-release mitochondrial protonophore; ETC, electron transport chain; HFD, high-fat diet.

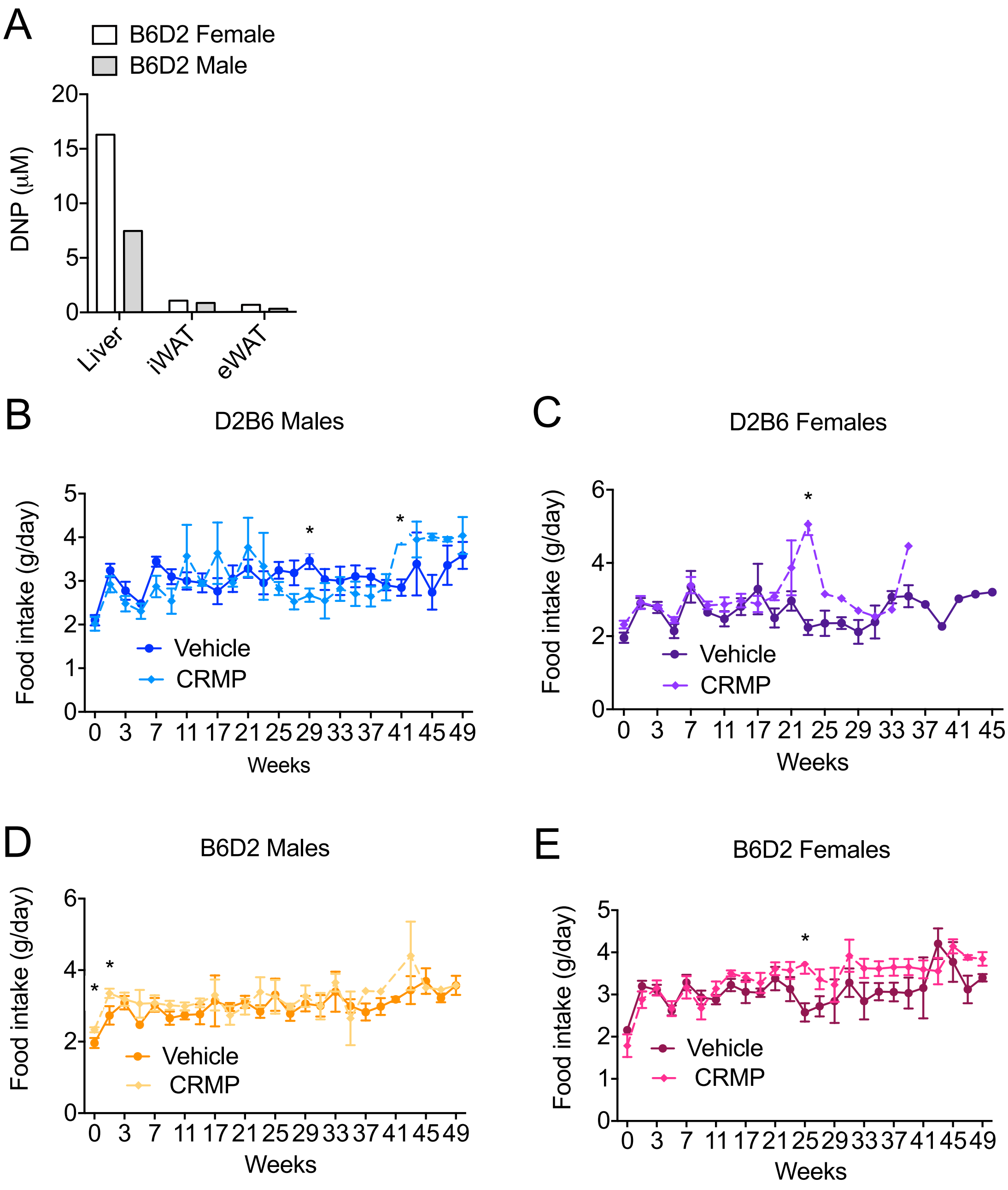

Figure S3: Food intake in aged HFD-fed male and female mice. (**A**) Tissue DNP concentrations in 6 h fasted 94-104 week-old HFD-fed D2B6 male and female mice treated with CRMP (10 mg/[kg-day]) for 3 months. *n* =1 per group. (**B–E**) Food intake trajectories of 94-104 week-old male and female F1 offspring of DBA/2J (D2) and C57BL/6J (B6) mice fed a HFD (45% fat) or HFD containing CRMP (10 mg/[kg-day]) for the remainder of their lives. In panels (**B–E**) data are presented as mean +/- SEM. *n* = 1129 per treatment group. **P*< 0.05 by two-sided unpaired Student's *t*-test compared to vehicle control. Abbreviations: C57BL/6J (B6)**;** CRMP, controlled-release mitochondrial protonophore; DBA/2J (D2); DNP, 2-4 dinitrophenol; HFD, high-fat diet.

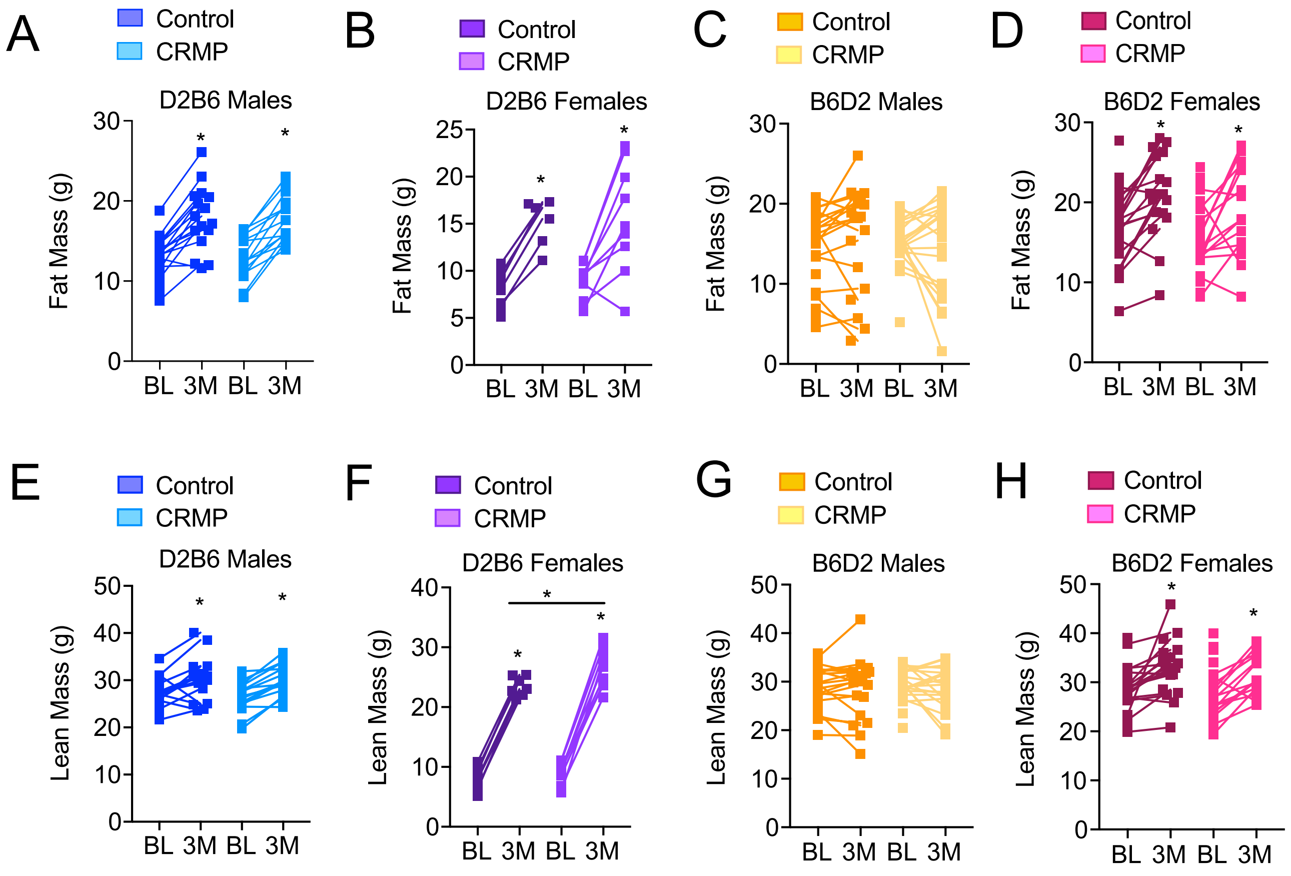

Figure S4: Sex and strain-specific effects of CRMP on lean and fat mass. Fat mass (**A–D**) and lean mass (**E**–**H**) in male and female F1 offspring of DBA/2J (D2) and C57BL/6J (B6) mice fed a HFD (45% fat; baseline [BL]) or HFD containing CRMP (10 mg/[kg-day]) for 3 months (3M). In all panels, *n* = 7-28 per group. Data are presented as mean +/- SEM. *P<0.05 by two-sided unpaired or paired Student's *t-test* (**A–B**, **D–H**) or Wilcoxon matched-pairs signed rank test (**C**). Abbreviations: C57BL/6J (B6)**;** CRMP, controlled-release mitochondrial protonophore; DBA/2J (D2); HFD, high-fat diet; F, fat mass; L, lean mass.

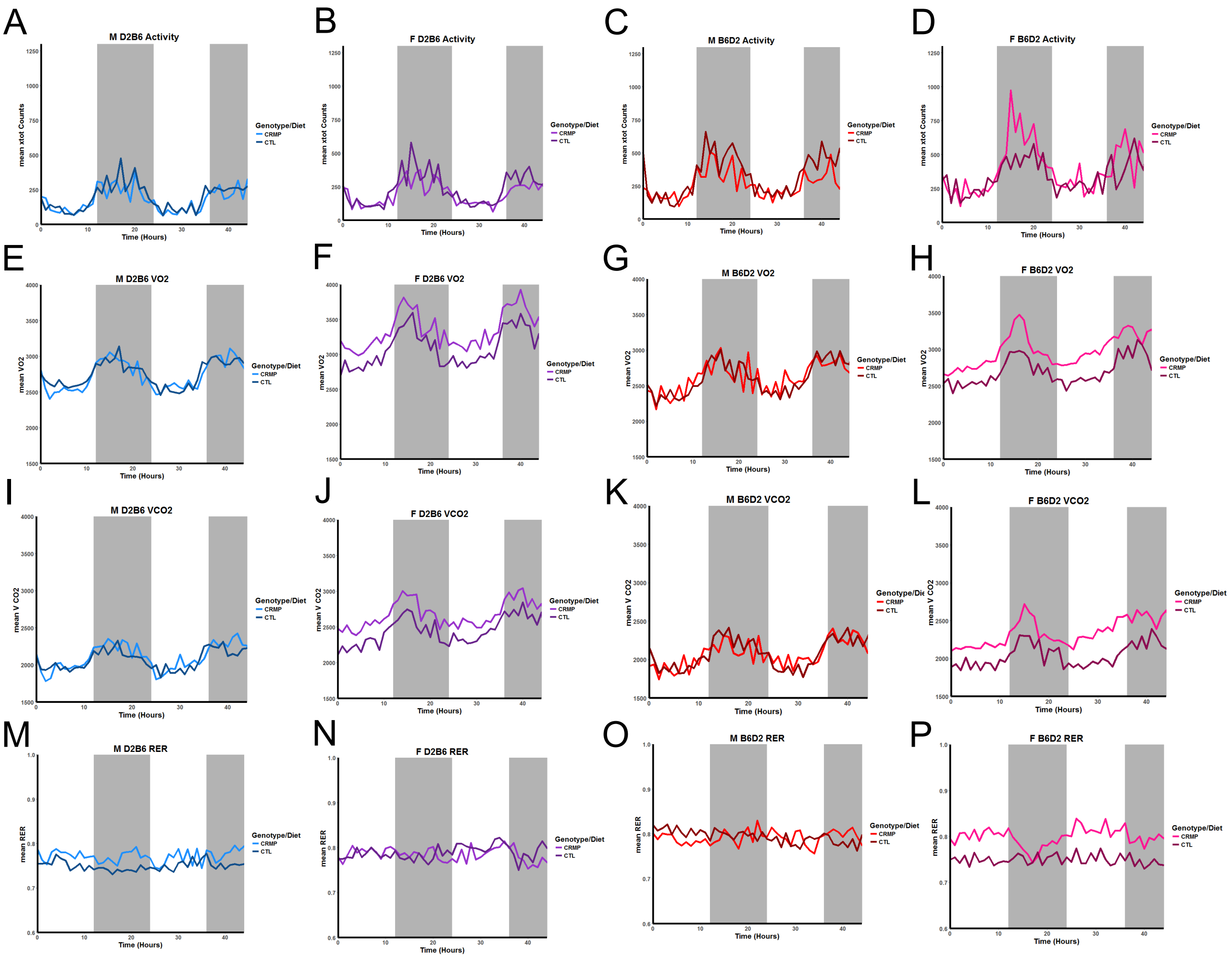

Figure S5: Sex and strain-specific effects of CRMP on whole-body metabolic parameters. Locomotor activity (**A–D**), whole-body O_2 consumption (**E–H**), whole-body VO_2 production (**I–L**), and RER (**M–P**) in 94-104 week-old male and female F1 offspring of DBA/2J (D2) and C57BL/6J (B6) mice fed a HFD (45% fat) or HFD containing CRMP (10 mg/[kg-day]) for 3 months. Data are presented as mean +/- SEM. *n* = 4 per treatment group. Abbreviations: C57BL/6J (B6)**;** CRMP, controlled-release mitochondrial protonophore; DBA/2J (D2); HFD, high-fat diet; RER, respiratory exchange ratio.

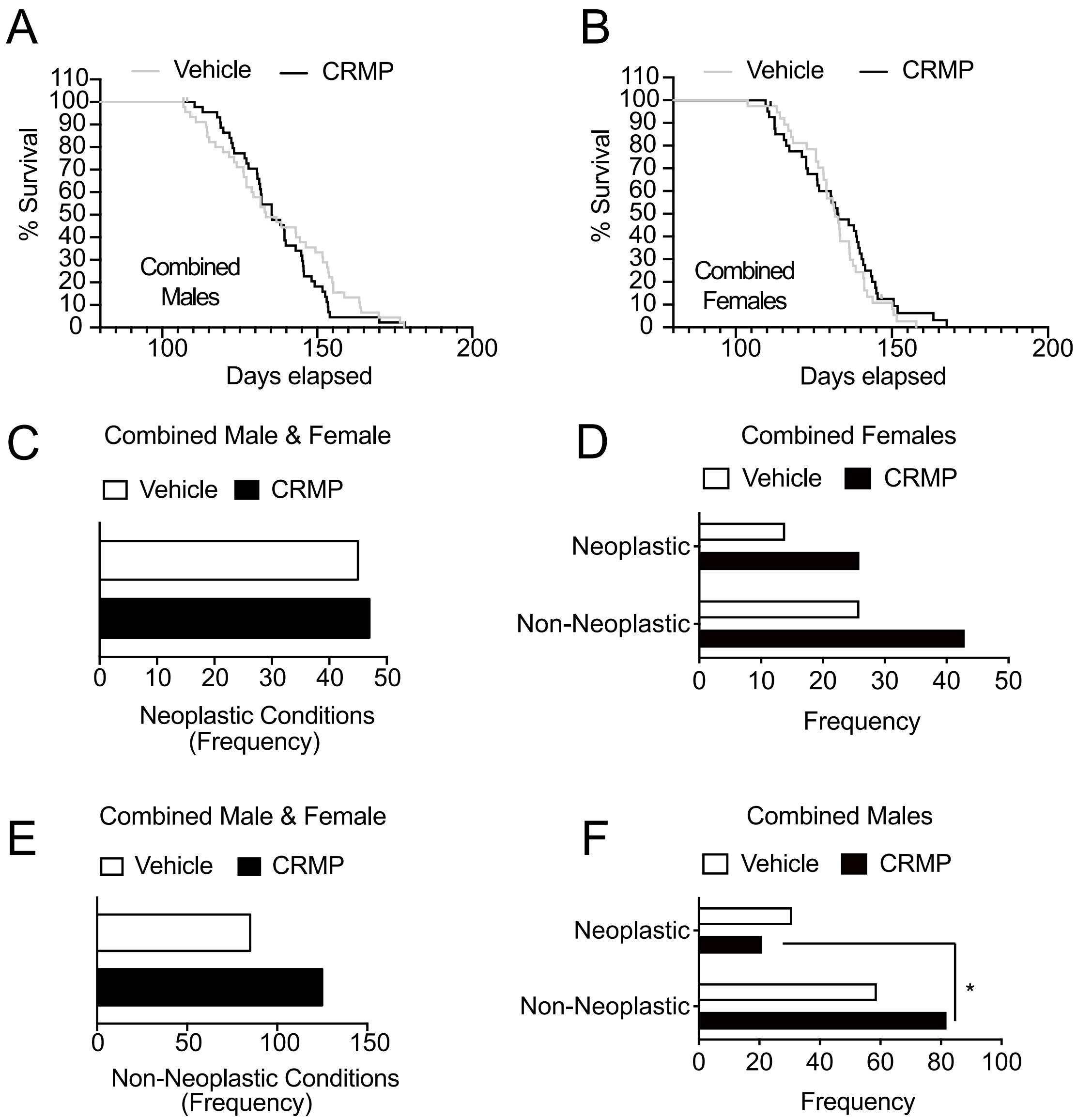

Figure S6: Sex-specific effects of CRMP on neoplastic and non-neoplastic disease incidence in HFD-fed mice. (**A–B**) Kaplan-Meier survival curves for D2B6/B6D2 males (**A**) and D2B6/B6D2 females (**B**) fed a HFD (45%) or HFD containing CRMP (10 mg/[kgday]). (**C**) Frequency of all neoplastic disease incidence in male and female mice treated as in (**A**). (**D**) Frequency of neo- and non-neoplastic disease incidence in female mice treated as in (**A**). (**E**) Frequency of all non-neoplastic disease incidence in male and female mice treated as in (**A**). (**F**) Frequency of neo- and non-neoplastic disease incidence in male and female mice treated as in (**A**). n = 30-31 per treatment group. *P<0.05 by Fisher's exact test (neoplastic vs non-neoplastic disease). Abbreviations: C57BL/6J (B6)**;** CRMP, controlled-release mitochondrial protonophore; DBA/2J (D2); HFD, high-fat diet.

Table S1: Survival statistics for mice stratified by gender and strain.

	Males						Females					
	B6D2				Combined		B6D2				Combined	D2B6
D2B6												
	Vehicle	CRMP	Vehicle	CRMP	Vehicle	CRMP	Vehicle	CRMP	Vehicle	CRMP	Vehicle	CRMP
**Log-rank (Mantel-Cox) test**												
Chi square	0.07974		1.386		0.6776		1.725		1.977		0.763	
df	1		1		1		1		1		1	
P value	0.7776		0.2391		0.4104		0.1890		0.1597		0.3824	
**Median survival**	143	145.4	132.4	131.9	133.4	135.4	131.4	132.9	135.6	130.4	131.9	132.7
Ratio (and reciprocal)	0.9833	1.017	1.004	0.9962	0.9858	1.014	0.9892	1.011	1.04	0.9616	0.9935	1.007
95% CI of ratio	0.5111-1.892	0.5286-1.957	0.5858-1.72	0.5814-1.707	0.6506-1.494	0.6695-1.537	0.5831-1.678	0.5958-1.715	0.4417- 2.449	0.4084-2.264	0.6338-1.558	0.6419-1.578
**Hazard Ratio (logrank)**	A/B	B/A	A/B	B/A	A/B	B/A	A/B	B/A	A/B	B/A	A/B	B/A
Ratio (and reciprocal)	1.096	0.9126	0.709	1.41	0.8342	1.199	1.405	0.712	0.5747	1.74	1.216	0.8225
95% CI of ratio	0.5701-2.106	0.4748-1.754	0.4-1.257	0.7956-2.5	0.5418-1.284	0.7786-1.846	0.8202-2.405	0.4157-1.219	0.2389- 1.383	0.7232-4.187	0.7728-1.913	0.5228-1.294
**Number of rows**	38	38	53	53	92	92	57	57	21	21	79	79
# of blank lines	19	19	27	26	47	46	29	28	11	10	41	39
# censored subjects	0	2	0	0	0	2	1	1	0	0	1	1
# deaths/events	19	17	26	27	45	44	27	28	10	11	131.9	132.7
